# The impact of biological properties of bioactive glasses on enamel during artificial erosive tooth wear

**DOI:** 10.2340/biid.v13.45670

**Published:** 2026-03-23

**Authors:** Dimitrios Dionysopoulos, Petros Mourouzis, Avraam Konstantinidis, Lambrini Papadopoulou, Kosmas Tolidis, Robert G. Hill

**Affiliations:** a Department of Operative Dentistry, Faculty of Dentistry, School of Health Sciences, Aristotle University of Thessaloniki, Thessaloniki, Greece; b Department of Civil Engineering, Division of Structural Engineering, Faculty of Engineering, Aristotle University of Thessaloniki, Thessaloniki, Greece; c Department of Mineralogy-Petrology-Economic Geology, School of Geology of Aristotle University of Thessaloniki, Thessaloniki, Greece; d Institute of Dentistry, Dental Physical Sciences Unit, Queen Mary University of London, London, United Kingdom

**Keywords:** bioactive glass, enamel, erosion/abrasion challenge, surface loss, surface hardness, air-abrasion

## Abstract

**Objectives:**

The aim of this study was to evaluate the protective effect of air-abrasion with two bioactive glasses (BAGs) on enamel surface against erosion/abrasion challenge.

**Materials and methods:**

Thirty human third molars were collected, and enamel specimens were prepared and randomly assigned to three groups (*n* = 10): control group, where specimens received no treatment and BAG groups, where enamel was air-abraded once for 10 seconds with BioMinF® and ProSylc™, respectively. The operational parameters were: air pressure 20 psi, powder flow rate dial 1 g/min and nozzle-surface distance 5 mm. The samples were submerged in a 0.01 M HCl solution for 2 minutes and then placed in a remineralizing solution for 2 hours (five times daily). At the end of each day, the samples were stored in the remineralizing solution for 14 hours. Thirty minutes after the initial and final erosive challenges of the day, an abrasion challenge was conducted using an electric toothbrush. Erosive tooth wear (ETW) was evaluated by measuring enamel surface loss, while surface hardness and roughness were measured to provide an indirect assessment of ETW-related changes. Additional insights into the bioactivity of the treatments were obtained by analyzing alterations in enamel morphology and composition. The data were statistically analyzed using one-way Analysis of Variance (ANOVA).

**Results:**

BAG treatments significantly reduced surface loss (38.7–46.7%) and increased surface hardness (6.3–8.9%) and roughness in enamel (*p* < 0.05). No significant differences were observed between the two BAG treatments (*p* > 0.05). Alterations in enamel surface morphology and composition were detected in the BAG groups compared to the control.

**Conclusions:**

Air-abrasion treatment with BAGs can provide a protective effect against early ETW, particularly under conditions that simulate gastroesophageal reflux disease (GERD)–related defects. Clinically, this implies that BAG air-abrasion may serve as a minimally invasive preventive treatment for patients at high risk of ETW, such as those with GERD.

## Introduction

Nowadays, there is an increased interest in bioactive materials, especially in health sciences [1]. The progress of technology offered the development of a variety of novel materials with improved bioactivity. Such materials include bioactive glasses (BAGs), which have been proposed for use in many clinical applications. In dentistry, BAGs are broadly used in clinical practice and have various indications such as preventive treatments against tooth erosion [2–4] and dental caries [5, 6], as well as therapy for tooth sensitivity [7–9], due to their ability to remineralize enamel and dentin surfaces through forming a calcium phosphate rich layer [10].

Specifically, BAGs are biocompatible silicate-based materials, containing calcium and phosphate in an amorphous matrix [3]. They elicit a distinct biological response at the interface, promoting the formation of a chemical bond between living structures and the material itself [11]. Their biocompatibility is attributed to the development of a biologically active hydroxycarbonate apatite layer that bonds with calcified tissues, such as bone and tooth tissues. One specific type of BAG, known as Bioactive Glass 45S5, which is a calcium-sodium-phosphosilicate, is widely used in dental applications due to its ability to promote remineralization and exhibit antibacterial effects. Additionally, it has been claimed that fluoride-containing BAGs (i.e. BioMinF®) are capable of forming fluorapatite [Ca_10_(PO_4_)_6_(OH)_(2–x)_F_x_] [12], which is less soluble (K_sp_≈10^–60^) compared to hydroxyapatite (HA) [Ca_10_(PO_4_)_6_(OH)_2_] (K_sp_≈10^–58^) and possibly more advantageous in terms of remineralization and tooth tissue repair [13]. Bioglass 45S5 (NovaMin®) is widely used in desensitizing toothpastes and professional prophylaxis powders, while BioMinF® is incorporated into preventive products such as remineralizing toothpastes and varnish-like formulations designed to enhance enamel resistance to acid challenges.

Meanwhile, gastroesophageal reflux disease (GERD) is a common chronic digestive disorder, affecting 10–20% of the population, and occurs when stomach acid (largely hydrochloric acid) and, occasionally, stomach contents flow back into the esophagus and may reach to the oral cavity [14]. This backflow of acidic contents can irritate and damage the lining of the esophagus, leading to a range of symptoms and potential complications including tooth erosion through a combination of acid exposure and regurgitation of stomach contents into the oral cavity. Regurgitated acids from the stomach present a very low pH (1–3) [15], which can trigger demineralization of the tooth tissues, increasing the risk of erosive tooth wear (ETW).

ETW is characterized as a chemical-mechanical process that results in the gradual loss of hard tissues of teeth, which is attributed to acids that do not originate from microorganisms [16]. The mechanism of ETW involves the demineralization of tooth surfaces, not only at the area where the tooth meets the erosive agent but also within a thin, partially demineralized layer just beneath the tooth surface, a process that is referred to as ‘near-surface demineralization’ [17]. These sub-surface lesions reduce hardness, making tooth tissues more susceptible to abrasive forces and result in a progression of ETW [18]. Research has shown that ETW is present in 24–48% of patients with GERD [19].

Dental treatment is designed to inhibit ETW in the early stages by applying preventive approaches, which primarily revolve around the use of bioactive compounds in different forms. The goal is to modify the tooth’s surfaces to decrease the susceptibility of HA crystals to acid-induced erosion, through buffering mechanisms [17, 20, 21]. Despite the potential benefits of preventive measures for dental erosion, their effectiveness unquestionably depends on patient adherence, thereby introducing a significant risk of potential failure [22]. In this context, it has been found that air-abrasion of enamel with particles of BAGs could be a useful in-office preventive treatment for reducing erosive activities [20, 21, 23–25] in combination with at-home regimens.

Therefore, the purpose of this in vitro study was to investigate the protective effect of air-abrasion with two BAG-containing powders BioMinF® and ProSylc™, on enamel against erosion/abrasion challenges simulating the conditions that induce early ETW in patients with GERD. Quantitative evaluation of ETW was conducted by measuring enamel surface loss using confocal microscopy, while qualitative assessment involved measuring changes in enamel surface roughness and hardness. Further insights into the bioactivity of the treatments were obtained by observing alterations in enamel morphology using scanning electron microscopy (SEM). Additionally, chemical analysis of apatite crystal formations was conducted using energy-dispersive X-ray spectroscopy (EDS).

The first null hypothesis of the study stated that enamel surface loss following air-abrasion treatments would not be different from the control group after the erosion/abrasion challenge. The second null hypothesis stated that between the two tested air-abrasion treatments, there would be no differences in enamel surface loss following the erosion/abrasion challenge.

## Materials and methods

### Experimental design

Two BAG materials were used in the current study: BioMinF® (referred to as BMF), which includes a fluoride-containing BAG and ProSylc™ (referred to as PSC), which contains Bioglass 45S5 (NovaMin®). ProSylc™ served as the positive control in this study, as it has been shown to be effective against enamel erosion in previous research [3, 4, 20, 21, 24]. The primary study factor was the enamel surface treatment, with three levels: (1) untreated enamel (negative control), (2) air-abrasion with BMF, and (3) air-abrasion with PSC (positive control). The response variables include enamel surface loss as the primary quantitative outcome, with surface hardness and surface roughness as secondary outcomes. In addition, qualitative analyses of enamel morphology and elemental composition were performed to further assess the bioactivity of the treatments. Furthermore, all experimental procedures, including specimen preparation, air-abrasion application, and erosion/abrasion challenges, were performed by a single calibrated operator. Where applicable, outcome assessments were conducted in a blinded manner to minimize potential bias. The technical characteristics of the tested materials are presented in Table 1.

**Table 1:** The technical characteristics of the materials used in the present study according to manufacturers.

Material	Active agent	Manufacturer	Composition
BioMinF® (BMF)	Calcium phospho-fluoro-silicate	Cera Dynamics Ltd Fountain Street, Fenton, Stoke-on-Trent, ST4 2HB, UK	36–40 mol% SiO_2_, 22–24 mol% Na_2_O, 28–30 mol% CaO, 4–6 mol% P_2_O_5_, 1.5–3.0 mol% CaF_2_
ProSylc™ (PSC)	NovaMin® (Bioglass 45S5)	Velopex, Harlesden, UK	Particle size: 30-60-90 μm, 45% SiO_2_, 24.4% CaO, 24.6% Na_2_O, 6% P_2_O_5_

### X-ray fluorescence analysis

X-ray fluorescence (XRF) was used for chemical analysis and the determination of chemical composition of the tested BAG powders. Elements with low atomic weight (H_2_, He and Li) cannot be identified with XRF; however, all the other elements can be identified. Bulk analyses of the specimens were determined by a S4-Pioneer (Bruker-AMS, Karlsruhe, Germany) wavelength dispersive spectrometer (XRF). The spectrometer was fitted with a rhodium (Rh) tube with five analyzing crystals, namely, LIF200, LIF220, LIF420, XS-55 and PET, and the detectors were a gas-flow proportional counter, scintillation detector or a combination of the two. Samples were analyzed at 60 kV and 45 mA tube-operating conditions [26]. The results of XRF analysis of the powder sample produced using the melting method are presented in Table 2, which shows, with acceptable matching, that the weight percentages are similar to standard weight percentages of PSC and BMF. The analysis for BMF revealed a slight lower content of SiO_2_ compared to manufacturer’s data. Additionally, fluorine has been under-detected, but fluorine is a very light element and on the limits of detection by XRF. Regarding PSC, SiO_2_ was in higher content, while CaO and Na_2_O were a bit lower. Despite these discrepancies, the results confirmed the production of bioglass with favorable weight percentages.

**Table 2:** Chemical analysis of the tested powders (wt%) using X-ray fluorescence analysis.

Compounds	BioMinF®	ProSylc™
SiO_2_	37.10	53.26
CaO	24.20	22.27
Na_2_O	28.56	21.17
P_2_O_5_	10.24	3.22
F	0.30	0.00
Total	100.40	99.91

### Preparation of BioMinF® powder for air-abrasion

The frit particles of BMF that were supplied were ground for 3 minutes using an automated mortar grinder (Retsch RM100, Haan, Germany). Afterwards, the powder was dried in a dry sterilizer at 125°C for 30 minutes and then sieved for 30 minutes using an automated sieve shaker (Octagon Digital 200, Endecotts, UK) fitted with a 100-μm sieve followed by a 60-μm sieve. The portion that passed through the 100-μm sieve but was retained on the 60-μm sieve (particles ranging between 60 and 100 μm) was collected. This resulting powder was stored in airtight containers with a silica gel pouch and wrapped in parafilm tape to maintain its quality [25]. To analyze the particle size, a confocal microscope (3D Optical Surface Metrology System Leica DCM8, Leica Microsystems CMS GmbH, Mannheim, Germany) was used because it permits non-destructive analysis, preserves the native particle geometry, and provides reliable size measurements in the micrometer range relevant to air-abrasion powders [25]. The hardness of the frit particles of BMF was measured using a nanoindentation tester (Hit 300, Anton Par TriTec SA, Corcelles, Switzerland) after incubation in acrylic resin and was 4.39 ± 1.03 GPa.

### Preparation of enamel specimens

The Ethical and Research Committee of Aristotle University of Thessaloniki approved this study (No. 77/6-12-2022), which was conducted in accordance with the ethical standards outlined in the 1964 Declaration of Helsinki and its subsequent amendments [27], while adhering to the policies of the local university. The patients included in this study provided their informed consent for the use of their teeth in research purposes. Participants were those attending the outpatient clinic during a 3-month period.

A total of 30 intact human third molars were collected and preserved in a 0.5% chloramine T solution at 6°C for a maximum period of 3 months. Immediately following extraction, any remaining soft tissues were carefully removed, and the teeth underwent cleaning with a slurry consisting of pumice and water. Subsequently, the tooth crowns were separated from the roots using a water-cooled diamond disc (Isomet, Buehler, Lake Bluff, IL, USA). Each specimen had approximate dimensions of 4 mm length and 4 mm width. Throughout the experiment, the specimens were not subjected to dehydration and were examined using an optical microscope (×10 magnification) to identify any surface structural defects. They were then randomly divided into three groups (*n* = 10) and embedded in acrylic resin (NT Newton AYCLIFFE Cold repair, Antalya, Turkey). The lingual surfaces were oriented upward during embedding since these surfaces are relatively flat and are most susceptible to erosive damage by gastric juice in patients with GERD.

To prepare the enamel surfaces for testing, they were ground and polished using a polishing machine (Jean Wirtz TG 250, Dusseldorf, Germany) operating at 200 rpm with water cooling (at a rate of 50 mL/min). This process involved gradually using 600-, 800-, 1000-, and 1200-grit silicon carbide abrasive papers (Struers, Copenhagen, Denmark) and a 0.4 μm alumina polishing suspension to achieve parallel and planar surfaces. Following polishing, the specimens were immersed in an ultrasonic bath (Euronda Spa, Montecchio Precalcino, Vicenza, Italy) for 5 minutes to remove any impurities and subsequently stored in distilled water at 37 ± 1°C for 24 hours.

### Experimental groups of the study

The study comprised three experimental groups (*n* = 10) as follows:

Group 1: This group served as the control of the study and did not undergo any treatment prior to the erosion/abrasion challenge.
Groups 2 and 3: The enamel surfaces in these groups were treated using an Aquacare^TM^ clinical air-abrasion unit (Velopex, Harlesden, UK). Group 2 specimens were air-abraded with BMF powder (Cera Dynamics Ltd, Fenton, UK), while Group 3 specimens were treated with PSC powder (Velopex, Harlesden, UK). Each specimen in Groups 2 and 3 underwent a 10-seconds air-abrasion pretreatment in wet mode, where the air stream was enveloped with a curtain of deionized water. The operational parameters were set as follows: air pressure at 20 psi (≈1.38 bar), powder flow rate dial set to 1 g/min, nozzle angle at 90°, nozzle-surface distance at 5 mm, and internal nozzle diameter at 900 μm [25, 28]. Air-abrasion was performed only once on the initial experimental day, precisely 1 hour before the first erosion/abrasion challenge. Subsequently, the specimens were promptly immersed in a fresh remineralizing solution at 37 ± 1°C. The composition of the remineralizing solution was as follows: 0.1029 g CaCl_2_·2H_2_O, 0.04066 g MgCl_2_, 0.544 g KH_2_PO_4_, 4.766 g HEPES buffer acid form, 2.2365 g KCl in 1000 mL distilled water, pH = 7 [29].

### ETW simulation

To obtain initial erosive enamel surfaces, the samples were treated by immersing them in a 0.01 M HCl solution for 30 seconds as described by Young et al. [30]. To mimic the erosive effect of gastric juice on enamel surfaces in patients with GERD, the samples underwent a 5-day erosive cycling procedure [25]. During each cycle, the samples were submerged in a 0.01 M HCl solution with a pH of 2.3 for 2 minutes at 25°C. Previously, the acidity of the solution was confirmed using a digital pH-meter (Orion Star^TM^ Series ISE Meter, Thermo Scientific, Beverly, USA). Afterward, the samples were rinsed with deionized water for 20 seconds and then placed in a remineralizing solution for 2 hours. This process was repeated four times daily (five cycles in total) by the same operator, and during this regimen, the remineralizing and acidic solutions were replaced after each cycle. At the end of each day, the samples were soaked in fresh remineralizing solution overnight (for 14 hours) at 37 ± 1°C.

**Figure 1:**
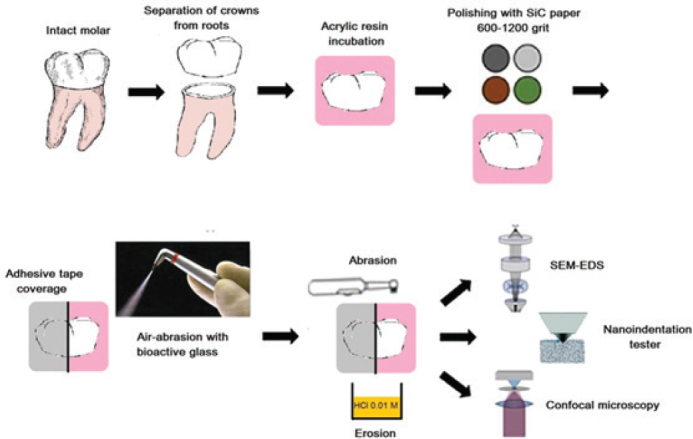
The workflow of the experimental protocol of the study.

Thirty minutes after the initial and final erosive challenges of the day, an abrasion challenge was conducted using a commercial oscillating-rotating electric toothbrush (Oral-B® iO, Braun, France) [31]. This meant that the abrasive challenge was carried out twice daily over a period of 5 days. The specific parameters for tooth brushing were set as follows: the toothbrush was loaded with a standardized force of 200 g (1.96 N) and featured a medium hardness and round toothbrush head. Each enamel specimen was brushed for 5 seconds during each abrasive challenge, totaling 10 seconds per day [20]. To ensure consistency, the electric toothbrush was securely placed in a specially designed apparatus that aligned the brush heads parallel to the surface of the specimens. The pressure applied was controlled by an electric system. The toothbrush head made direct contact with the specimen, and a slurry was created using a regular toothpaste (Colgate®Total, Colgate-Palmolive Company, Greece), which had a relative dentin abrasivity (RDA) value of 70. The composition of the toothpaste was as follows: water, glycerin, hydrated silica, PVM/MA copolymer, sodium lauryl sulfate, cellulose gum, aroma, sodium hydroxide, carrageenan, sodium fluoride (1450 ppmF^–^), triclosan, sodium saccharin, limonene and CI 77891. This slurry was formed by mixing toothpaste and deionized water in a 1:1 (w/w) ratio. The specimens were immersed in this slurry for 2 minutes while the brushing procedure was performed (5 seconds for each specimen). Following this, the specimens were rinsed with deionized water for 20 seconds, air-dried for 5 seconds, and then immersed in a remineralizing solution at 37 ± 1°C [32]. The workflow of the experimental protocol of the study is illustrated in Figure 1.

### Surface loss assessment

Before the air-abrasion treatment and erosion/abrasion challenge, half of the enamel surface on each specimen was covered with adhesive tape (Wonder® Tape, P.V.C. Electrical Tape). Following the erosion/abrasion challenge, surface loss measurements were conducted using a confocal microscope (3D Optical Surface Metrology System Leica DCM8, Leica Microsystems CMS GmbH, Mannheim, Germany) in profilometer mode. Once the adhesive tape was removed, four images (at ×20 magnification) were taken from the center of each specimen’s surface, covering an area of 0.660 × 0.877 mm^2^. The results of surface loss were obtained by evaluating the level of the formed step between the treated and untreated surface. For each image, five measurements were taken (50 μm apart), and the data were averaged and reported in μm.

### Evaluation of surface roughness changes

The surface roughness of the enamel specimens was assessed according to ISO 25178 standards (non-contact method). Measurements were taken both before and after the erosion/abrasion challenge with the same confocal microscope utilized for surface loss assessment. For each specimen, three images in every quadrant were captured (×20 magnification), covering an area of 1.3 × 1.6 mm^2^. The Leica Map software (Leica GmbH, Germany) was employed to collect data and determine the average surface parameters (Sa and Sdr) of each image. The values from the 12 images of each specimen were averaged, and the means of the surface parameters were calculated.

### Evaluation of surface hardness changes

Surface hardness of each enamel specimen was evaluated prior and after the erosion/abrasion challenge. Evaluation of enamel surface hardness was conducted using a nanoindentation tester (Hit 300, Anton Par TriTec SA, Corcelles, Switzerland). The type of the diamond indenter was Berkovich, the maximum load was 40 mN, the time to maximum load was 30 seconds, the approach speed 4000 nm/min, and the acquisition rate 10 Hz at quadratic loading. Ten indentations were carried out on the top surface of each specimen with 100 μm distance to each other, and the means of Martens hardness (HM) in N/mm^2^ were calculated with the Indentation Software Version 10.

### SEM and energy dispersive X-ray spectroscopy analyses

In each experimental group, three specimens (*n* = 3) were analyzed to identify changes in the enamel surface after the erosion/abrasion challenge and following the other testing procedures, which were not destructive to the specimens. The specimens were prepared according to the experimental protocol, mounted on aluminum stubs, and coated with a carbon layer of approximately 200 Å thickness using a vacuum evaporator (under low vacuum conditions). The examination was carried out using a scanning electron microscope (JEOL Ltd, JSM-840, Tokyo, Japan) with an accelerated voltage of 20 kV. Four photomicrographs were captured, one from each quadrant, and were magnified at ×1000 and at both secondary electron imaging (SEI) and backscattered electron imaging (BEC). Additionally, EDS was employed to evaluate potential changes in the mineral composition of the enamel surfaces after the erosion/abrasion challenge.

### Statistical analysis

The results of the study were analyzed statistically with the use of SPSS Statistics 23.0 software (IBM Corp, ILL, Chicago, USA). The sample size of each testing was determined using the formula $n=\frac{z_{\alpha /2}^{2}\times s^{2}}{e^{2}}$ where *n* = sample size, *z*_α/2_ = 1.96 (according to the normal distribution) for α = 0.05 (significance level), *s* = standard deviation, and *e* = sampling error. The data were initially tested for normality and homogeneity, using Shapiro–Wilk and Levene tests, respectively. Surface loss, hardness and roughness data of the specimens were statistically analyzed using one-way ANOVA, separately. Tukey’s post-hoc test was used to identify any statistical differences at a level of significance *a* = 0.05. The mineral composition of enamel was analyzed using the Wilcoxon signed rank and Kruskal-Wallis tests with significance preset at *p* < 0.05.

## Results

### Surface loss outcomes

**Figure 2:**
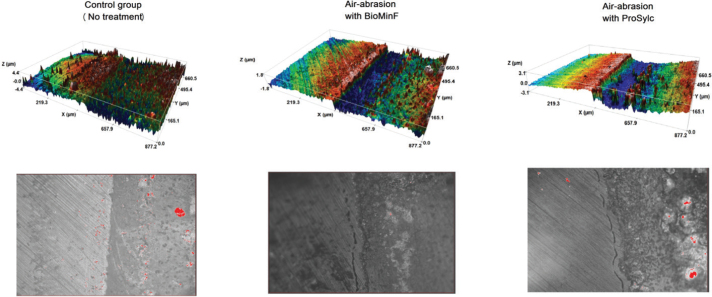
Topographic 3D surface maps (upper) and confocal microscopy images (lower) of representative specimens of each experimental group at ×20 magnification. The left side of the specimens was covered with the adhesive tape and remained untreated. Surface loss compared to the baseline surface can be observed and calculated after the erosion/abrasion challenge.

Means and standard deviations of surface loss (μm) of the experimental groups of the study after erosion/abrasion challenge are presented in Table 3. Following the erosion/abrasion challenge, all experimental groups exhibited surface loss to different extents. The untreated group presented the highest surface loss (*p* < 0.035), followed by air-abraded groups (2 and 3), which did not differ from each other (*p* = 0.753). Figure 2 shows a representative topographic surface map (×20 magnification) and surface analysis of one specimen of each experimental group.

**Table 3:** Means and standard deviations of surface loss (μm) of the experimental groups of the study after the erosion/abrasion challenge.

Treatments	Mean surface loss (μm)	Decrease (%) compared to control
No treatment	5.12 ± 1.39^A^	-
Air-abrasion with BioMinF®	2.73 ± 1.41^B^	46.7%
Air-abrasion with ProSylc™	3.14 ± 1.72^B^	38.7%

The decrease (%) in surface loss of the tested treatment compared to the control group is also presented. Same uppercase superscripts in the column indicate no significant differences between the groups (*p* > 0.05).

### Surface roughness outcomes

**Figure 3:**
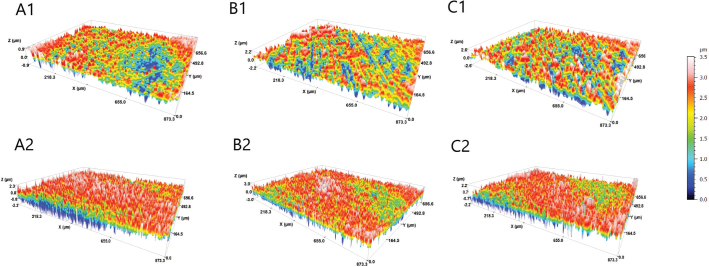
Topographic 3D surface maps of representative specimens (magnification ×20) of each experimental group before (1) and after (2) erosion/abrasion challenge. A: control group; B: BioMinF treatment; C: ProSylc treatment.

Means and standard deviations of surface roughness expressed in Sa (μm) and Sdr (%) of the experimental groups of the study before and after erosion/abrasion challenge are presented in Table 4. Representative topographic surface maps (×20 magnification) of specimens’ surfaces of each experimental group are illustrated in Figure 3. After erosion/abrasion challenge, all the experimental groups presented significantly higher surface roughness parameters (*p* < 0.05), as indicated in Figure 3 with red color. Following erosion/abrasion challenge, the control group presented significantly lower Sa values than the air-abraded groups (*p* < 0.001), which did not differ from each other (*p* = 0.478). In addition, the air-abraded groups exhibited significantly higher Sdr values compared to the control group (*p* < 0.001), while they did not significantly differ from each other for Sdr (*p* = 0.129).

**Table 4:** Means and standard deviations of surface roughness (Sa, μm and Sdr, %) of the experimental groups of the study before and after erosion/abrasion challenge.

Treatments	Sa (μm) before erosion/abrasion	Sa (μm) after erosion/abrasion
No treatment	0.32 ± 0.11^Aa^	0.67 ± 0.22^Ab^
BioMinF®	0.36 ± 0.07^Aa^	0.97 ± 0.07^Bb^
ProSylc™	0.39 ± 0.09^Aa^	0.96 ± 0.09^Bb^
Treatments	Sdr (%) before erosion/abrasion	Sdr (%) after erosion/abrasion
No treatment	0.15 ± 0.10^Aa^	21.09 ± 11.22^Ab^
BioMinF®	0.12 ± 0.08^Aa^	46.72 ± 29.77^Bb^
ProSylc™	0.13 ± 0.08^Aa^	40.48 ± 29.70^Bb^

Same uppercase superscripts in columns indicate no significant differences among experimental groups (*p* > 0.05). Same lowercase superscripts in rows indicate no significant differences between before and after erosive/abrasion challenge (*p* > 0.05).

### Surface hardness outcomes

Means and standard deviations of Martens Hardness (HM) in N/mm^2^ are shown in Table 5. Surface hardness was significantly reduced in the control group after erosion/abrasion challenge (*p* = 0.017), whereas in air-abraded groups, no significant difference was observed between them (*p* = 0.253).

**Table 5:** Means and standard deviations of Martens hardness (HM) in N/mm^2^ of the experimental groups of the study before and after erosion/abrasion challenge.

Treatments	HM before erosion/abrasion	HM after erosion/abrasion	HM decrease %
No treatment	2662 ± 184^Aa^	2278 ± 243^Ab^	14.4%
BioMinF®	2732 ± 236^Aa^	2587 ± 242^Bb^	5.3%
ProSylc™	2690 ± 203^Aa^	2472 ± 222^Bb^	8.1%

Same uppercase superscripts in columns indicate no significant differences among experimental groups (*p* > 0.05). Same lowercase superscripts in rows indicate no significant differences between before and after erosion/abrasion challenge (*p* > 0.05).

### SEM observations

**Figure 4:**
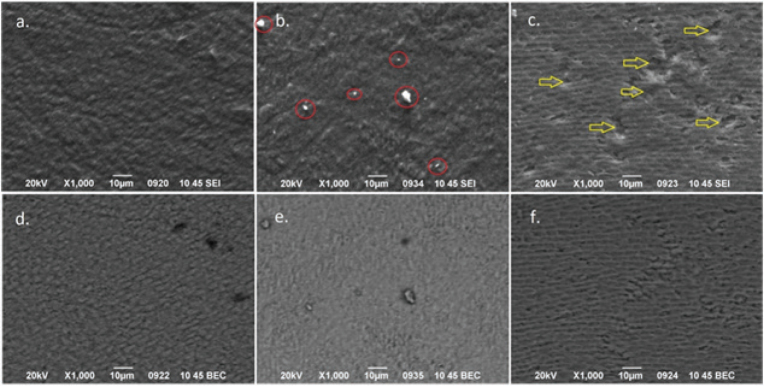
Representative SEM images in secondary electron imaging SEI (a–c) and BEC (d–f) mode of each experimental group after the erosion/abrasion challenge at ×1000 magnification. Red circles indicate remaining bioactive glass particles and yellow arrows craters possibly induced by air-abrasion. Control group (a and d), BioMinF® (b and e) and ProSylc™ (c and f). SEM: scanning electron microscopy; SEI: secondary electron imaging; BEC: backscattered electron imaging.

Representative SEM images in SEI and BEC modes at ×1000 magnification of the enamel surface of the specimens for each experimental group after erosion/abrasion challenge are shown in Figure 4. These images revealed slight alterations in enamel surface morphology in the air-abrasion groups compared to the control group. Specifically, sparse deposits of inorganic particles were detected on the enamel surface of air-abraded specimens, likely originating from powders containing BAG particles (BMF and PSC), as confirmed by EDS analysis. These residual particles were more abundant in the BMF group. The specimens in the air-abrasion groups exhibited rougher surfaces, consistent with the Sdr measurements. Notably, craters (yellow arrows), possibly caused by particle collisions with the enamel surface during the air-abrasion procedure appeared more frequently in the PSC group. In all experimental groups, enamel prisms were visible due to the erosion/abrasion challenge applied.

### Energy dispersive X-ray spectroscopy analysis

Means and standard deviations of the elemental content (wt%) of the enamel surface after the treatments of each experimental group are shown in Table 6. EDS analysis revealed slight differences in composition of the enamel surfaces among the experimental groups. In particular, in the air-abrasion groups with BAG particles, sodium (Na) and silicon (Si) were detected in increased amounts compared to the control group, indicating the presence of BAG particles on the enamel surface. Additionally, in BMF group, a low content of fluoride (F) was also present, as it is included in the composition of the particles. The identification of the detected residual particles was confirmed with EDS analysis.

**Table 6:** Means and standard deviations of elemental content (wt%) of enamel surface after the erosion/abrasion challenge of each experimental group of the study.

Elements	No treatment	BioMinF®	ProSylc™
Ca	30.09 ± 3.82^a^	28.70 ± 4.44^a^	28.88 ± 3.39^a^
P	18.27 ± 2.11^a^	17.68 ± 3.50^a^	17.40 ± 2.71^a^
Si	0.00 ± 0.00^a^	6.15 ± 0.73^b^	8.76 ± 1.14^c^
Na	0.44 ± 0.10^a^	4.18 ± 0.42^b^	6.05 ± 1.09^c^
Mg	0.64 ± 0.12^a^	0.31 ± 0.06^b^	0.20 ± 0.06^c^
Cl	0.82 ± 0.15^a^	0.45 ± 0.09^b^	0.43 ± 0.05^b^
F	0.00 ± 0.00^a^	1.51 ± 0.04^b^	0.00 ± 0.00^a^
O	49.75 ± 3.59^a^	41.19 ± 3.08^b^	40.13 ± 4.00^b^

Different lowercase superscripts in rows indicate statistically significant difference between the groups (*p* < 0.05).

## Discussion

According to the results of the current study the first null hypothesis, which stated that enamel surface loss following air-abrasion treatments would not be different from the control group after the erosion/abrasion challenge, was rejected. Particularly, enamel surface loss was observed across all experimental groups following the erosion/abrasion challenge. This is due to the chemical activity of the HCl on enamel, which leads to demineralization of the tooth surface in combination with the abrasive forces that applied by the electric toothbrush and enhance surface loss. However, the specimens air-abraded with BMF and PSC exhibited significantly less surface loss (46.7 and 38.7%, respectively) compared to the control group. This finding is consistent with previous studies that reported beneficial effects against tooth erosion when air-abrasion treatments using powders containing BAGs were applied [2, 3, 20, 21, 25, 28].

BAGs are biocompatible silicate-based materials, containing calcium and phosphate in an amorphous matrix [3] engineered to interact with living tissues, promoting advantageous outcomes such as forming a bond between the glass and the surrounding tissue [33]. These biomaterials have a reactive surface that dissolves when in contact with tooth surfaces and saliva, a process that accelerates under acidic conditions. During dissolution, calcium and phosphate ions are released, increasing the pH and promoting the remineralization of demineralized enamel surface damaged by acid exposure, as seen in dental erosion [4]. When BAGs come into contact with bodily fluids such as saliva, they initiate a series of processes, including the development of a HA or carbonate hydroxyapatite (HCA) layer on the surface of the tissues [34–36]. While it takes several hours for this apatite layer to develop on teeth, it can act as a protective barrier against erosive agents, thereby increasing resistance to enamel demineralization [4]. Consequently, the lower surface loss that was observed in the present study in the air-abraded groups with BAG-containing powders may be attributed to this mechanism.

In a previous study [20], air-abrasion treatment with PSC, following the same clinical protocol, removed approximately 2.5 μm of enamel tissue from erosive tooth surfaces. This suggests that PSC particles may possess greater hardness than enamel, and that acid-treated enamel, being significantly softer than intact enamel, is more susceptible to removal. This finding aligns with the hardness of BAG 45S5 particles (4.68 GPa) measured by Thompson and Hench [34], which exceeded that of the tested enamel (≈3.5 GPa). In the present study, air-abrasion with BMF and PSC resulted in similar surface loss and roughness, indicating that BMF may exhibit comparable hardness. Indeed, the hardness of BMF was found in the current study 4.39 ± 1.03 GPa, confirming this assumption. Consequently, the enamel removal from the air-abrasion pretreatment with BMF and PSC was included in the total surface loss, suggesting that the surface loss caused by the subsequent erosion/abrasion challenge may have been lower.

Between the tested air-abrasion treatments no significant differences were observed in the reduction of enamel surface loss. Consequently, the second null hypothesis was accepted. This is in contrast with a previous report [25], which demonstrated that air-abrasion with BMF presented statistically significant lower enamel surface loss compared to that with PSC after erosive challenge with artificial gastric juice. The main difference between these two studies was that in the older one there was no abrasion challenge that may crucially influence the results of enamel wear in acidic conditions [16]. In that study the authors interpreted this difference by the discrepancies in the composition of the tested powders.

PSC contains Bioglass 45S5, a calcium sodium phospho-silicate marketed under the registered trade name NovaMin®. In general, glasses are amorphous materials lacking long-range structural order, unlike crystalline materials, which exhibit a regular array of atomic positions repeated in space, indicating long-range order [37]. Bioglass 45S5 differs from conventional glasses by containing less silica and higher concentrations of calcium and phosphorus. Specifically, PSC comprises 45 wt% SiO_2_, primarily in the form of polymeric silica chains, and a calcium-to-phosphorus (Ca/P) ratio of approximately 5:1, which enables the formation of apatite crystals. Additionally, calcium and silica ions serve as crystallization nuclei [38]. The high Ca/P ratio in Bioglass 45S5 is critical for bonding to bone and tooth tissues, as lower Ca/P ratios do not facilitate such bonding [39].

BMF, in comparison to Bioglass 45S5, contains fluoride and has significantly higher phosphate content, as it was confirmed by XRF analysis in the present study (F: 0.3 wt% and P_2_O_5_: 10.24 wt%). Increasing the phosphate content accelerates apatite formation, increases the quantity of apatite produced, and significantly reduces the pH rise during dissolution [40–42]. Consequently, BMF is believed to promote faster and greater apatite crystal formation than Bioglass 45S5. This aligns with SEM observations of a previous investigation [25], which revealed a larger quantity of apatite crystals on BMF-treated specimens, completely covering the enamel surface. BMF contains fluorine within the glass in the form of sodium calcium fluorine (F-Ca/Na_(n)_) species. Even small amounts of fluoride in the glass can accelerate apatite formation, favoring the development of fluorapatite over HCA. Fluorapatite is less soluble, dissolving at a lower pH (~4) compared to HA (~5), thereby potentially improving enamel resistance to erosion [43]. However, increasing the fluoride content in the glass further reduces the pH rise and may promote the formation of calcium fluoride (CaF_2_) at the expense of fluorapatite [42].

In the current study, air-abrasion treatment with BMF showed similar enamel surface loss and hardness compared to PSC, suggesting that the presence of fluorine in this content in the composition may not have significant clinical benefits regarding ETW. Nonetheless, the surface chemical analysis of the BMF treated enamel surface showed a significant fluorine content of 1.51 weight percent. This value is much higher than the XRF determined fluorine content of the glass at 0.3%. This suggests that fluorine is being concentrated in the enamel surface. If the enamel surface was entirely fluorapatite a fluorine content of approximately 3.8% would be expected. A fluorine content of 1.5% represents an approximately 40% conversion to fluorapatite from HA. Taha et al. [13] found evidence of FAP formation on enamel surfaces using ^19^F magic angle nuclear magnetic resonance spectroscopy after air abrasion with a different fluorine containng BAG and immersion in an artificial saliva. The formation of fluorapatite would be expected to reduce acid erosion, but it does not have a significant influence. It may be that the low pH used of 2.3 is sufficient to dissolve both fluoridated apatite and HA present in the enamel surface.

As previously mentioned, the primary protective mechanism of BAGs against tooth erosion is the formation of an HCA layer on the tooth surface, which acts as a barrier to erosive agents. In earlier studies, mineral precipitations covering the enamel surface were observed following erosive challenges. These studies showed that BAG particles became embedded in the treated enamel surface, promoting remineralization [4, 20]. The precipitations were described as dumbbell-like crystallites arranged in flower-like clusters [4, 23, 25]. In contrast, the current investigation did not reveal such characteristic mineral precipitations in SEM observations. This could be attributed to the abrasive forces applied during tooth brushing simulation, which may have removed or altered the newly formed HCA layer. However, SEM-EDS analysis confirmed that BAG particles remained embedded in the enamel surfaces. Furthermore, a higher surface hardness was recorded in air-abraded specimens compared to the untreated, suggesting remineralization activity on the enamel. This finding aligns with other studies that have also reported increased surface hardness of tooth surfaces following BAG treatments [4, 20, 24, 44, 45].

Following the erosion/abrasion challenge, all experimental groups in the study showed a decrease in surface hardness. This reduction may be attributed to mineral loss from the enamel surface caused by the artificial erosive agent. However, the tested air-abrasion treatments mitigated the drop in surface hardness similarly to one another when compared to the control group. These differences may be due to the remineralizing effect and the protective layer formed by BAG particles deposited on the enamel surface [23, 46, 47].

Changes in enamel surface roughness following an erosion/abrasion challenge reflect the interaction between acidic agents, abrasive forces, and the tooth surface. This interaction can lead to the removal of inorganic compounds or modifications in apatite crystals, depending on factors such as duration of exposure, acidity of the erosive agent, magnitude of applied forces, hardness of the bristles of the toothbrush, oral conditions, and tooth substrate [48]. In the present study, all experimental groups exhibited a significant increase in surface roughness after the erosion/abrasion challenge. Specifically, the air-abrasion treatment groups showed a similar increase in surface roughness parameters, though greater than that of the control group. This suggests that the tested air-abrasion treatments contributed to the increase in surface roughness, likely due to the impact of BAG particles on the enamel, leading to tissue removal, as well as the presence of embedded BAG particles, as confirmed by SEM observations. These findings coincide with previous studies investigating the effects of air-abrasion on tooth surfaces [6, 13]. A previous report [21] highlighted the importance of powder selection in dental air-abrasion procedures, noting that excessive abrasion of sound enamel increases its susceptibility to acid erosion, primarily due to increased surface roughness. However, BAG powders were found to be less invasive than alumina (Al_2_O_3_) powder due to their lower hardness, supporting their potential use in preventing dental erosion [21].

It is important to mention that enamel HA is highly ordered, more crystalline, and mechanically superior, while that formed by BAGs is less crystalline, and more bioactive. Both involve chemical precipitation but in vivo the concentrations of calcium and phosphate are controlled by cellular processes and protein templating [49]. This makes BAG-formed HA suitable for biomedical applications, such as protection of enamel from erosion, as the results of the present study indicated. Stoichiometry, acidity and solubility of HA can be estimated through the Ca/P ratio. Higher Ca/P ratio yields lower acidity and solubility and vice versa [50]. Furthermore, enamel HA is highly crystalline, well-organized into long, tightly packed apatite rods, which gives enamel its high mechanical strength. On the other side, BAG-formed HA is poorly crystalline or even amorphous when first precipitated, while over time, it can become more crystalline, but it never reaches the same level of organization, particularly in terms of crystal orientation, as enamel HA [50]. As a result, it is softer and more fragile due to its lower crystallinity and structural disorganization. This might be the reason for not detecting any HA-layer formed by BAG after tooth brushing simulation.

Undoubtedly, stannous fluoride (SnF_2_) is considered the gold standard for managing ETW and has frequently been used as a positive control in previous studies [20, 25, 51, 52]. It has been reported that following a severe erosive challenge, such as exposure to gastric regurgitation, SnF_2_ forms a protective barrier layer on the pellicle-coated tooth surface, which adheres to eroded tissues and offers substantial protection [51, 52]. However, SnF_2_ is not designed for in-office application and requires consistent daily use over 1–2 weeks. Therefore, we deemed it inappropriate to compare the effectiveness of an at-home preventive treatment like SnF_2_ with in-office procedures such as BAG air-abrasion treatments.

In the current investigation, a clinically relevant model was utilized to assess the susceptibility of enamel to HCl-induced erosion, followed by abrasion using an electric toothbrush. The erosive challenge involved a solution with a pH of 2.3, consistent with previous studies [53]. While the pH of gastric juice typically ranges from 0.9 to 1.5, salivary buffering and dilution prevent the pH of oral cavity from dropping below 1.5. Additionally, under clinical conditions, the presence of the acquired enamel pellicle and proteins such as mucin and statherin modulate enamel interactions with acids and abrasives, reducing wear effects [53]. Since the pH in the oral cavity remains low for no more than 2 minutes after a reflux episode [54], similar exposure times were used in this study to better simulate in vivo conditions. While in vitro models using HCl and electric toothbrushes provide controlled and reproducible conditions to study erosion/abrasion, they cannot fully replicate the complexity of the oral environment. Future studies should complement these findings with in situ or in vivo research to confirm real-world effectiveness and clinical relevance.

BAG–based air-abrasion represents a minimally invasive, non–technique-sensitive preventive approach that may be easily incorporated into routine clinical workflows, particularly for patients at high risk of ETW. From a cost–benefit perspective, the use of BAG powders may offer a favorable balance by potentially reducing the need for more extensive restorative interventions, thereby lowering long-term treatment costs and chairside time. In addition, the materials investigated are already commercially available and used in preventive and prophylactic dental products, which support their feasibility for clinical implementation without significant additional investment in equipment or training.

## Conclusions

Within the limitations of this in vitro study, it was concluded that both tested air-abrasion treatments using powders containing BAGs (BMF and PSC) were effective in protecting enamel from erosive wear compared to the control group. In particular, the BAG-treated groups exhibited lower surface loss and higher surface hardness than the enamel specimens that did not receive any treatment. No significant differences were found between the BAG treatments. Further studies and clinical evidence are needed to confirm the effectiveness of these in-office preventive treatments and determine their suitability for inclusion in tailored preventive programs against ETW, particularly in patients with conditions such as GERD.

## Data Availability

The data presented in this study are available upon request from the corresponding author.
